# A randomized trial of fish oil omega-3 fatty acids on arterial health, inflammation, and metabolic syndrome in a young healthy population

**DOI:** 10.1186/1475-2891-12-40

**Published:** 2013-04-08

**Authors:** Martin Root, Scott R Collier, Kevin A Zwetsloot, Katrina L West, Megan C McGinn

**Affiliations:** 1 Department of Nutrition and Health Care Management, Appalachian State University, Boone, NC, 28608, USA; 2 Department of Health, Leisure, and Exercise Science, Appalachian State University, Boone, NC, 28608, USA; 3 5231 Cypress Palms Lane, Tampa, FL, 33647, USA; 4 17 Salem Acres, Weaverville, NC, 28787, USA

**Keywords:** Fish oil, Inflammation, Metabolic syndrome, Pulse wave velocity, Randomized trial

## Abstract

**Background:**

Long chain omega-3 fatty acids from fish oils (O3) are known to have beneficial effects on a number of vascular risk factors in at-risk populations. The effects of a highly bioavailable emulsified preparation on an overweight young adult population are less well known.

**Methods:**

Young adults, age 18–30, with body mass indices (BMIs) greater than 23 (average = 28.1) were administered 1.7 g of O3 per day (N = 30) or safflower oil placebo (N = 27) in an emulsified preparation (Coromega, Inc.) for 4 weeks in a double-blind randomized design. Blood was drawn and anthropometric measurements taken before and after dosing. Hemodynamic measures (central pulse wave velocity, augmentation index, and aortic systolic blood pressure), inflammatory cytokines (IL-6, IL-8, IL-10, and tumor necrosis factor-α), red blood cell and plasma phospholipid fatty acid profiles, fasting serum lipids, glucose, and C-reactive protein were measured.

**Results:**

Red cell and plasma phospholipid eicosapentaenoic acid and docosahexaenoic acid concentrations increased over the four weeks of dosing in the O3 group. Dosing with O3 did not affect central pulse wave velocity, augmentation index, or aortic systolic blood pressure. None of the five American Heart Association metabolic syndrome components improved over the dosing period. None of the inflammatory cytokines, C-reactive protein, or lipids (total or LDL cholesterol) improved over the dosing period.

**Conclusions:**

No salutary effects of O3 were observed in hemodynamic, metabolic syndrome criteria or inflammatory markers as a result of this relatively short period of administration in this relatively overweight, but healthy young adult cohort.

## Background

Metabolic syndrome has become highly prevalent in the U.S. and has a strong impact on the development of future vascular ailments including Type II diabetes mellitus and cardiovascular diseases. This increase can be partially attributed to recent changes in the American diet that negatively affect body weight and arterial health [1].

Arterial stiffness is associated with metabolic syndrome and is a predictor of cardiovascular events [2]. Pulse wave velocity (PWV) and augmentation index (AIx) are measurements of arterial distensibility [3]. Reducing dietary saturated fats and increasing omega-3 polyunsaturated fats, especially from fish (O3), have long been known to improve vascular health and may improve measures of arterial stiffness [4]. Dangardt et al. reported supplementation with O3s improved vascular function and lowered the severity of inflammation among the obese [5]. However, Mackay, et al. found fish oil supplementation among those with or at risk of heart disease and receiving aspirin and statin therapy had no effect on pulse wave velocity [6]. O3s have also been found to improve obesity-induced metabolic syndrome through regulating chronic inflammation [7]. These include C-reactive protein (CRP), interleukin-6 (IL-6), and tumor-necrosis factor-α (TNF-α) [8].

While previous researchers have focused their studies on disease risk factors in older at-risk populations [9,10], the present study sought to investigate the effects of O3 supplementation in a relatively overweight college-aged population. The pathologies of vascular diseases have been observed and recorded in the very young. Earlier intervention in those at early risk may show greater promise in longer-term benefits. Researchers have found that younger populations have responded favorably to O3 dosing during weight loss in reducing triglycerides, leptin, insulin, insulin resistance, and blood pressure and increasing ghrelin concentrations [11-14]. In addition, younger adults tend to consume relatively low amounts of fish and have relatively low levels of O3s in blood and tissues [15]. This might make this group especially responsive to the beneficial effects of even low doses of O3. Others have also found high PWV and Aix values among obese adolescents [16]. Thus, this study investigated whether O3s improved arterial health and inflammatory responses, both being indicators of risk for metabolic syndrome and ultimately, heart disease, diabetes, and stroke, in a healthy young relatively overweight population.

## Methods

### Subjects

Healthy 18–30 year old men and women were recruited from the Appalachian State University student population. 430 responded. Respondents were sent a survey to determine inclusion and exclusion criteria, including self-reported height and weight and weekly exercise engagement. Of those initial surveys, 343 were returned. Sixty subjects were invited to participate in the study based on the following inclusion criteria: body mass index (BMI) >23 and engagement in regular exercise of no more than 3 times per week. Subjects were also not taking any cardiovascular medications or fish oil supplements, not allergic to fish, or with a history of diabetes, heart disease or a stroke, and did not consume fish more than twice per week. The research protocol was approved by the Institutional Review Board of Appalachian State University.

### Study design

The initial treatment and control groups consisted of 30 and 27 participants, respectively. Three subjects who were invited to enter the study failed to attend the first clinical visit. Randomization was performed from a random digits table. The subjects recruited for this four-week, double-blind study completed two visits to the clinic. At the first clinical visit, anthropometric measurements, including height, weight, and waist circumference, and seated blood pressure were measured by a single trained technician. Hemodynamic testing included central PWV and AIx, which is further used to determine aortic pressure [17]. Hemodynamic testing was performed by 3 different trained technicians. A fasting blood sample was drawn for blood lipids, red blood cell and plasma phospholipid fatty acid profiles, high sensitivity C-reactive protein (hsCRP), blood glucose, and inflammatory cytokines. A Health History Questionnaire was completed at home and returned within the first week. Participants were requested to comply with their reported usual exercise habits of three or less times per week during the study period.

At the conclusion of the first clinical visit, subjects were supplied with four weeks’ worth of O3 or placebo oil single-dose packets. Treatment consisted of 4 weeks of supplementation with O3, 350 mg eicosapentaenoic acid (EPA) and 230 mg docosahexaenoic acid (DHA) per single-dose packet, while the placebo was 1.0 g of safflower oil per packet. The oils were emulsified products (Coromega, Inc., Vista, CA) provided in three premeasured packets; one packet to be opened and consumed at breakfast, two packets at dinner. Protocol compliance was monitored with a check sheet that participants kept to record the supplements they consumed. A second clinical visit occurred at the end of the intervention period of four weeks and included a second round of clinical measures, blood draws and hemodynamic testing as before.

### Measurements

Height and weight were determined using a Healthometer (Jarden Corp, Rye, NY) stadiometer and scale. Waist circumference was measured manually with a tape measure. Blood pressure was measured using a manual sphygmometer according to AHA guidelines. Blood lipids, glucose, and CRP were measured at the local hospital laboratory.

Hemodynamic and vascular testing was performed with a manual blood pressure cuff and sphygmomanometer and the Sphygmacor Cardiovascular Management System. Applanation tonometry (SphygmoCor, Inc., Sydney, Australia) was used to derive the range of central arterial indices, including PWV and AIx [17]. The same trained technician preformed each measurement and our laboratory technicians have an intra-class correlation coefficient of 0.96 with a coefficient of variation (%CV) of <4.0%.

Serum lipids, glucose, and hsCRP were measured on a Dimensions RXL instrument from Siemens, Inc. Total cholesterol was measured by polychromatic endpoint technique using oxidase, peroxidase and esterase with a%CV of 1.82%. HDL cholesterol was measured by the accelerator selective detection method (direct measure polymer-polyanion) with a 0.92%CV. Triglycerides were measured by enzyme immunoassay with bichromatic endpoint with 3.60%CV. LDL cholesterol was measured by direct measure enzyme immunoassay with bichromatic endpoint with 2.72%CV. Glucose was measured by hexokinase with bichromatic endpoint with 3.50%CV. Finally, hsCRP was measured with colorimetric immunoassay with bichromatic endpoint with 2.1%CV.

Levels of inflammation protein targets were measured using the Meso Scale Discovery® Multi-Spot® Assay System. In this multiplex electrochemiluminescent ELISA, IL-6, IL-8, IL-10, and TNF-α concentrations were detected on spatially distinct spots in single wells on 96-well plates. Sample analyses were performed in duplicate. Intra-assay coefficient of variability between assay plates was 11% for IL-6, 5% for IL-8, 8% for IL-10, and 7% for TNF-α.

Fatty acid analysis was performed essentially according to the method of Lands, et al [18]. Briefly, sample lipids were extracted by the method of Bligh and Dyer [19]. Lipid fractions were separated on thin layer chromatography. Appropriate samples were then transesterified with boron trifluoride and extracted. Methyl esters were separated and quantified with a Shimadzu capillary gas chromatograph with flame-ionization detection. Authentic standards and internal standards were used. The %CVs for the long-chain omega-3 fatty acids were under 5%.

### Statistical analysis

Repeated measures general linear models were used to determine treatment effects. Chi Square and Student t-tests were used to determine differences between treatment groups at baseline. Analysis was performed with SPSS v.18 (SPSS, Inc. Chicago, Illinois). A power calculation was made to estimate group size *a priori*. A total of 30 subjects were required to give us adequate statistical power at a p < 0.05 for the outcome variables of PWV and AIx. Based on existing data from our laboratory, the estimated sample size of 30 subjects gives us an effect size of 0.92 and 0.88, respectively, with an alpha set at 0.05. Test-retest reliabilities in our laboratory for these 2 dependent variables are 0.98 and 0.97, respectively.

## Results

Of the 57 men and women who entered the study, 51 completed the intervention. Six participants did not return for the second clinical visit, four from the O3 group and two from the placebo group. Of those that completed the study, 41 returned the compliance check sheets, 19 from the placebo group and 22 from the fish oil group. Of those, 100% of the placebo participants consumed greater than 85% of their supplements while 86% of the fish oil participants consumed greater than 85% of their supplements.

Anthropometric measurements and blood samples were collected during the initial clinical visit. Table 1 summarizes baseline information collected. Subjects chosen were at risk of metabolic syndrome with blood pressure measurements and waist circumferences slightly higher than normal, particularly for women. There were no statistically significant differences in baseline measurements between the treatment groups.

**Table 1:** Baseline information of study subjects (percent or mean ± standard deviation)*

**Variable**	**Placebo group**	**Omega-3 group**
N	27	30
Age (years)	20.4 ± 2.1	21.4 ± 2.9
Female	38%	48%
Body mass index (kg/m^2^)	27.9 ± 2.9	28.5 ± 3.3
Systolic blood pressure (mmHg)	125 ± 7	124.5 ± 7
Waist circumference, females (cm)	86 ± 10	90 ± 11
Waist circumference, males (cm)	92 ± 8	96 ± 6
Triglycerides (mmol/L)	1.01 ± 0.56	1.01 ± 0.52
HDL-cholesterol (mmol/L)	1.22 ± 0.32	1.20 ± 0.26
Serum glucose (mmol/L)	4.7 ± 0.4	4.7 ± 0.4
Activity level (number times exercise per week)	3.0 ± 0.9	3.0 ± 0.9
Current Smokers	25%	8%

* None of the groups were statistically significantly different at p < 0.05.

**Figure 1:**
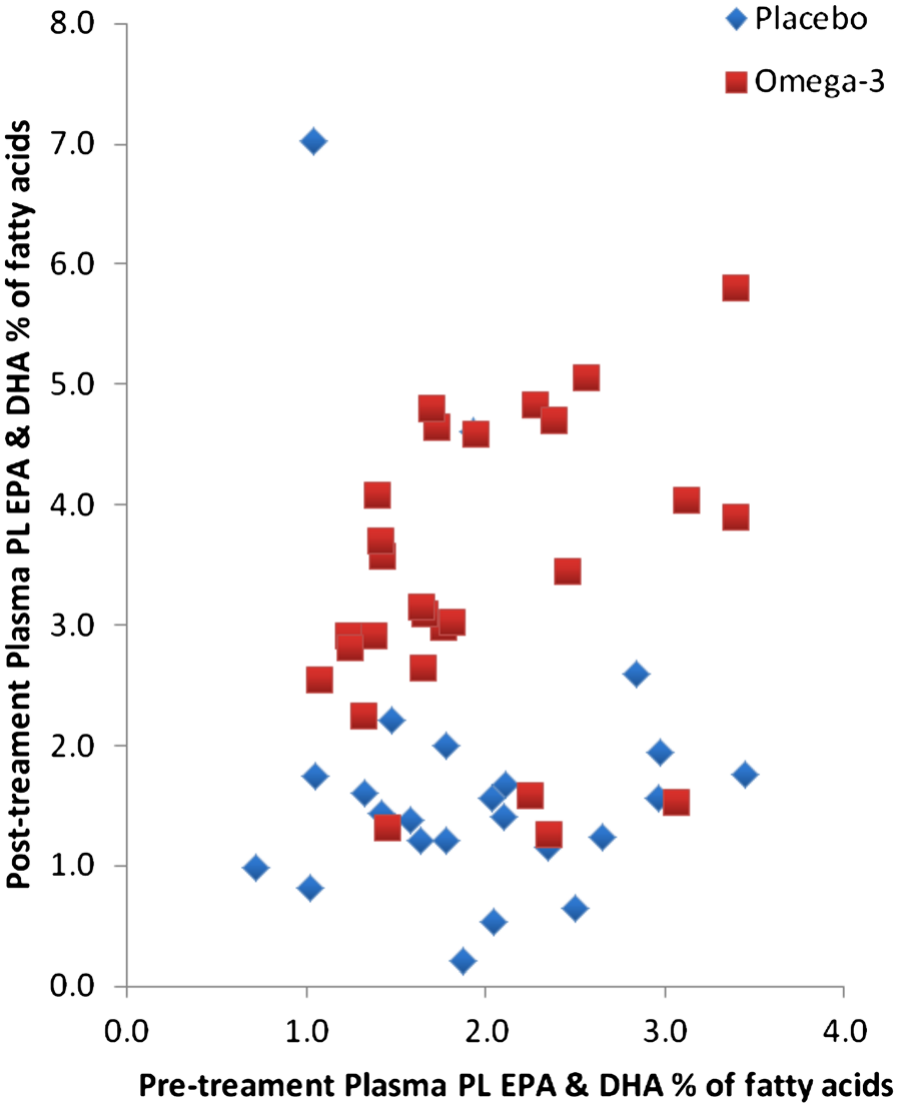
Effect of fish oil supplementation on plasma phospholipid combined EPA and DHA levels as percent of total fatty acids before and after treatment.

Measurements were also taken four weeks later, after O3 supplementation. Figure 1 shows the effect of O3 dosing for four weeks on plasma phospholipid EPA and DHA. Plasma levels increased significantly (p < 0.001).

Plasma and red blood cell phospholipid fatty acid outcomes are displayed in Table 2. Significant increases are noted for both EPA and DHA and their combination in both samples. In the plasma the combined EPA and DHA increased about 80% and in the red blood cells the combined fatty acids increased about half as much.

**Table 2:** Effect of fish oil supplementation on plasma and red blood cell phospholipid fatty acids*

**Markers**	**Placebo**		**Omega-3**		**Treatment difference**	**S.E. of difference**	**P-value of difference**
	**Before**	**After**	**Before**	**After**			
Plasma PL% EPA	0.332	0.400	0.329	1.225	+0.832	0.170	p < 0.001
Plasma PL% DHA	1.623	1.370	1.618	2.157	+0.762	0.281	p = 0.008
Plasma PL% EPA&DHA	1.95	1.77	1.95	3.38	+1.61	0.408	p < 0.001
RBC% EPA	0.314	0.373	0.366	1.026	+0.606	0.108	p < 0.001
RBC% DHA	2.930	3.082	3.169	4.169	+0.708	0.245	p = 0.019
RBC% EPA&DHA	3.244	3.455	3.535	5.195	+1.314	0.298	p = 0.002

* Repeated measure general linear models. PL = phospholipid, EPA = eicosapentaenoic acid, DHA = docosahexaenoic acid.

The effects of supplementation on components of the metabolic syndrome, plasma lipids, vascular measures, and cytokines are shown in Table 3. The daily treatment dose of 1.7 g of O3 for four weeks had no effect on any of these measures compared to the safflower placebo. Not shown are BMI, waist, total cholesterol, serum glucose, IL-8, and IL-10, all of which also showed no effect.

**Table 3:** Effect of fish oil supplementation on arterial measures*

**Markers**	**Placebo**		**Omega-3**		**Treatment difference**	**S.E. of difference**	**P-value of difference**
	**Before**	**After**	**Before**	**After**			
Aortic systolic blood pressure (mmHg)	112.1	110.0	110.8	111.1	+2.54	1.70	0.91
Central PWV (m/s)	7.44	6.61	7.49	7.43	+0.76	0.70	0.26
Augmentation Index (%)	10.8	7.4	10.9	11.6	+4.00	2.73	0.47
SBP (mmHg)	125.7	124.5	124.5	125.1	+1.9	1.7	0.26
LDL cholesterol (mmol/L)	2.41	2.39	2.47	2.48	-0.01	0.15	0.81
Triglycerides (mmol/L)	0.88	0.99	0.91	0.91	-0.099	0.145	0.83
HDL-cholesterol (mmol/L)	1.225	1.164	1.204	1.189	+0.004	0.050	0.74
TNF-α (pg/mL)	4.90	4.52	5.31	5.70	+0.14	0.08	0.75
IL-6 (pg/mL)	0.87	0.77	0.99	1.12	+0.21	0.19	0.51
CRP (nmol/L)	20.7	21.8	17.3	18.7	+0.12	0.34	0.53

* Repeated measure general linear models. PWV = Central pulse wave velocity, BMI = Body Mass Index, SBP = Systolic Blood Pressure. TNF-α = Tumor Necrosis Factor-α, IL-6 = Interleukin-6, CRP = C - reactive protein. Triglycerides, TNF-α, IL-6, and CRP are log transformed; means expressed as geometric means; differences are expressed as log values.

## Discussion

In this study we investigated the effect of O3 on relatively overweight but healthy young adults. We found no effect of 4 weeks of dosing with 1.7 grams per day on arterial hemodynamic measures, components of the metabolic syndrome, serum lipids, or measures of chronic inflammation. Yet, we were able to verify a change in O3 status in serum and red cell phospholipids with O3 dosing.

Emulsifying fish oils can enhance digestion and absorption of the fatty acids. Raatz, et al. investigated emulsified fish oil absorption compared with capsular triglyceride fish oil supplements in humans throughout a 48-hour observation period. A single dose (350 mg EPA and 230 mg DHA) of the emulsified product resulted in enhanced absorption of total O3 compared with the capsular supplement. Although we gave a relatively low dose, Raatz, et al. have shown that this material is absorbed more quickly and maybe more completely than fish oil in tablets [20]. Figure 1 shows that the O3 treatment increased plasma O3 EPA and DHA, despite four apparent non responders in the treatment group and two with increased EPA and DHA in the placebo group.

Arterial stiffness is associated with metabolic syndrome [2]. PWV and pulse pressure are measures positively associated with aortic stiffening, also measured by AIx [3,21]. Sjoberg, et al. introduced 2 g, 4 g, and 6 g of fish oil supplementation per day into the diets of overweight or obese adults for 12 weeks. Improvement in arterial distensibility, as measured by PWV, was only found to be significant only at the highest dose of 6 g of fish oil per day [22]. Chong, et al. reported a significant improvement in PWV and AIx, in healthy adults immediately after a long chain O3 PUFA-rich meal containing 4.7 g of DHA and EPA [4]. However, Sanders, et al. recently found that 1.8 g of EPA and DHA daily over 12 months did not improve arterial stiffness among slightly overweight but relatively healthy middle aged subjects in England [23]. Thus, using a comparable dose over a greater duration than in our study within an older age group yielded similar outcomes.

A number of studies have been conducted on the association between O3 intake and the development of the metabolic syndrome. The present study found that fish oils did not have an effect on components of the metabolic syndrome in overweight young adults. Pederson, et al. found that supplementation with 1.5 g of O3 per day for 16 weeks significantly lowered systolic blood pressure and raised HDL cholesterol in overweight adolescents [24]. Also, supplementation with O3 over a 12-week period significantly lowered serum glucose levels [25].

Inflammation is also recognized as having a significant relationship with metabolic syndrome [26]. Dietary patterns poor in O3 may cause an excessive production of pro-inflammatory cytokines and CRP, while causing a lower production of anti-inflammatory cytokines, all recognized as contributing to the inflammation associated with metabolic syndrome and cardiovascular events [26,27]. Dangardt, et al. executed an intervention of 1.2 g of O3 supplements per day on obese adolescents for 3 months having an average BMI of 33.8, compared to our group average BMI of 28.1. Results showed a significant decrease in TNF-α and IL-6 levels, but no significant change in the serum levels of CRP, IL-8 or IL-10 [5]. Low CRP levels have also been observed in Yup’ik Eskimos, a population who have mean daily intakes of DHA and EPA ranging from 2.4 to 3.7 g. In this population, CRP blood levels were inversely related to the intake of fish oils; however, there was no relationship found with IL-6 levels and fish oil intake [28]. Our results, in addition to those found in past studies, may further demonstrate the need for a longer intervention period, a higher treatment dose and an at-risk population selection for treatment to observe the desired results in inflammatory markers.

Overall, our present investigation found little effect of emulsified fish oil on components of the metabolic syndrome, inflammatory cytokines, or hemodynamic measures of arterial health. Other studies suggest that higher doses of fish oil coupled with a longer intervention period executed in a more unhealthy population may be needed to manifest positive and significant results from O3 intervention. Strengths of our study include the bioavailability of the emulsified supplement, clear evidence for incorporation of O3 doses into blood phospholipids, the young age of the participants, and the multiplicity of the endpoints, specifically the hemodynamic markers and the inflammatory cytokines. However, some limitations to the study are apparent. These include the short length in time of the dosing and the relatively low dose compared to other more recent studies.

## Conclusions

The administration of a highly bioavailable emulsified preparation of fish oil O3 measurably increased plasma and red blood cells EPA and DHA in four weeks. However, no beneficial changes were observed in the markers that were measured for hemodynamic, metabolic syndrome, or inflammatory effects as a result in this relatively healthy overweight young adult cohort. More at-risk populations may benefit more from this intervention.

## Abbreviations

CRP: C-reactive protein; DHA: Docosahexaenoic acid; EPA: Eicosapentaenoic acid; IL-6: Interleukin-6; IL-8: Interleukin-8; IL-10: Interleukin-10; O3: Fish oil omega-3 fatty acids; TNF-α: Tumor necrosis factor-alpha.

## Competing interests

Martin Root received funding for this research from the Dyson Foundation and the supplements from Coromega, Inc. The other authors declare that they have no competing interests.

## Authors’ contributions

MR was the Principal Investigator. He directed the study, the data analysis and the drafting of the manuscript. He finalized and submitted the manuscript. SC directed the arterial function testing and data interpretation and hosted study site. KZ conducted the inflammatory marker analysis and data interpretation. KW wrote the initial manuscript and performed the data analysis. MM managed the trial, recruiting subjects, scheduling both subjects and researchers and coordinating data collection. All authors reviewed and contributed to the final version of the manuscript.
